# Host, vector, and parasite dynamics: exploring intrinsic and extrinsic factors shaping tick‐borne filarial nematode transmission

**DOI:** 10.1002/brv.70146

**Published:** 2026-02-12

**Authors:** Oluwaseun D. Ajileye, Gabriel L. Hamer, Sarah A. Hamer, Guilherme G. Verocai, Jessica E. Light

**Affiliations:** ^1^ Ecology and Evolutionary Biology Program Texas A&M University College Station TX 77843 USA; ^2^ Department of Ecology and Conservation Biology Texas A&M University College Station TX 77843 USA; ^3^ Department of Entomology Texas A&M University College Station TX 77843 USA; ^4^ Department of Veterinary Integrative Biosciences Texas A&M University College Station TX 77843 USA; ^5^ Department of Veterinary Pathobiology College of Veterinary Medicine and Biomedical Sciences, Texas A&M University College Station TX 77843 USA

**Keywords:** biological transmission, filarial nematodes, microfilaria periodicity, midgut barrier, ticks, tick‐borne pathogens, vector competence

## Abstract

Tick‐borne filarial nematodes are a complex and understudied group of parasites that rely on ticks for transmission in vertebrates. This review examines how intrinsic and extrinsic factors may influence the successful transmission of filarial nematodes in tick vectors, drawing insights from extensively studied haematophagous dipteran vector systems. We explore how different tick life stages contribute to potential nematode transmission to the host and the influence of abiotic factors on nematode survival and development within ticks. Understanding nematode life stage periodicity and enhancement phenomena in tick‐borne filarial systems is equally important for determining transmission dynamics and co‐infection patterns. Also important to nematode transmission are vertebrate host movement and tick feeding patterns, which create opportunities for parasite exchange across ecological environments. Knowledge gaps include the physical and molecular mechanisms of transmission, the potential influence of tick genetic variation on vector competence, immune responses across different tick–filarial associations, and epidemiological studies of host and tick patterns of nematode infection. Comprehensive empirical data are also needed to model transmission patterns, including temperature and humidity thresholds for nematode development and survival, field‐based tick infection rates, and how tick immune responses affect these processes. Understanding these factors requires integrating expertise from multiple disciplines and adopting an ecosystem‐based perspective that considers the interconnected nature of vertebrate hosts, vectors, and environment in the transmission of filarial nematodes.

## I. INTRODUCTION

Understanding vector‐borne pathogen transmission is crucial for predicting and controlling pathogens and diseases. Filarial nematodes (Nematoda: Filarioidea) are parasitic helminths that have evolved complex life cycles involving arthropod vectors (i.e. intermediate hosts) and vertebrate hosts (i.e. definitive hosts), often causing a variety of diseases across diverse vertebrate species, including humans, domestic animals, and wildlife (Anderson, 2000; Bain, 2002). Throughout this review, we use the term ‘filarial’ rather than ‘filarioid’ to refer to these nematodes. While both terms appear in the literature, ‘filarial’ is more commonly used in medical, veterinary, and public health contexts (particularly in neglected tropical disease literature), whereas ‘filarioid’ is typically used in strict taxonomic and systematic discussions. Given our focus on vector–pathogen transmission dynamics and implications for animal and human health, we employ the broader term ‘filarial’ throughout this review. While mosquitoes, biting flies, and fleas are the best‐known vectors of filarial nematodes, ticks (Acari: Ixodida) are biological vectors of several filarial nematode species. However, tick‐borne filarial nematode transmission remains poorly understood despite their global distribution and potential impact on humans and domestic and wild animals (Anderson, 2000; Orihel & Eberhard, 1998). Additionally, vector competence – the ability of an arthropod to acquire, support the development of, and transmit a pathogen – has rarely been investigated for tick–filarial nematode systems (Ajileye, Verocai & Light, 2025). This research gap exists largely due to methodological challenges: laboratory studies require simultaneous maintenance of both ticks and appropriate vertebrate hosts, extended observation periods (often months) to accommodate the prolonged feeding behaviour of ticks and the slow development of filarial larvae, and specialized containment facilities. Despite these challenges, understanding the unique biological characteristics of ticks that may influence filarial nematode development and transmission dynamics is crucial for understanding the role of helminth parasites on human and animal health and predicting how environmental changes might alter host–vector–parasite relationships.

Tick‐borne filarial nematodes belong to the family Onchocercidae, primarily within a closely related group of genera (supported by molecular analyses; Lefoulon *et al*., 2015), including *Acanthocheilonema*, *Cercopithifilaria*, *Cruorifilaria*, *Monanema*, and *Yatesia*. Filarial nematodes require arthropod intermediate hosts to complete their life cycle, developing from immature microfilariae stages to infective larvae (L3 stage). While obtaining a blood meal, arthropod hosts such as ticks transmit, or vector, the infective larvae to vertebrate hosts where the filarial nematode life cycle continues. Filarial nematodes have been detected in ticks collected from diverse vertebrate hosts representing several taxonomic groups, including rodents, carnivorans, and wild and domesticated ungulates (Ajileye *et al*., 2025). While filarial nematodes have been documented within tick species globally, experimental studies of biological transmission – where the parasite undergoes development through all larval life stages within the vector and is transmitted to a vertebrate host – have been demonstrated for only a limited number of filarial–tick associations (Table 1). Vector competence, or the ability of an arthropod to transmit filarial nematodes, requires further investigation for most associations (Baltazard, Chabaud & Minou, 1952; Bain *et al*., 1978; Petit *et al*., 1988; Yates & Lowrie, 1984) (for a comprehensive review, see Ajileye *et al*., 2025).

**Table 1:** Reported tick vectors of filarial nematodes, their life stages of acquisition, development, and transmission, and their developmental periods. ‘Multiple stages’ is used where acquisition, development, or transmission can occur during multiple life stages. ‘Not specified’ indicates that filarial worms have been detected in a tick species, but the life stage was not described. ‘Unknown’ indicates that the tick life stage used for acquisition, development, or transmission is unknown.

Filarial nematodes	Tick vectors	Acquisition stage(s)	Development stage(s)	Transmission stage(s)	Development time (microfilariae to infective L3 stage)	References
*Acanthocheilonema*						
*A. dracunculoides*	*Rhipicephalus sanguineus* s.l. (Ixodidae)	Multiple stages	Nymphs, complete to L3; adults, partial to early L3 only	Nymphs	~15 days	Olmeda‐García *et al*. (1993); Olmeda‐García & Rodríguez‐Rodríguez (1994)
*A. viteae*	*Ornithodoros tartakovskyi* (Argasidae)	Multiple stages	Multiple stages	Multiple stages	~30 days	Londono (1976)
	*O. moubata* (Argasidae)	Multiple stages	Multiple stages	Multiple stages	~30 days	Singh *et al*. (1988)
*Cercopithifilaria*						
*C. bainae*	*R. sanguineus s.l*. (Ixodidae)	Nymphs: experimental and observations; Larvae (likely)	Adults, transstadial development to L3 occurs post‐moult in adults	Adults	25–30 days	Brianti *et al*. (2012)
*C. grassi*	*R. sanguineus s.l*. (Ixodidae)	Not specified	Not specified	Not specified, but L3 found in ticks	>30 days	Otranto *et al*. (2012b)
*C. rugosicauda*	*Ixodes ricinus* (Ixodidae)	Larvae; nymphs	Nymphs; adults	Nymphs; adults	56–67 days	Winkhardt (1980); Ramos *et al*. (2013b)
*Cherylia*						
*C. guyanensis*	*I. ricinus* (Ixodidae)	Not specified	Not specified	Not specified	~30 days	Bain *et al*. (1985)
*Cruorifilaria*						
*Cruorifilaria* sp.	*Amblyomma romitii*, *A. cajennense* (Ixodidae)	Unknown	Unknown	Unknown	Unknown	Binetruy & Duron (2023)
*Monanema*						
*M. globulosa*	*Haemaphysalis leachi* (Ixodidae)	Not specified	Not specified (intracellular development noted)	Adults: presumed ticks dissected 30–60 days post‐feeding	~30–60 days	Bianco *et al*. (1983)
*M. marmotae*	*I. cookei* (Ixodidae)	Larvae; nymphs: nymphs are noted as more efficient at acquiring microfilariae	Adults: transstadial‐intracellular development in fat body, epidermal cells and haemocoel	Adults	~30 days	Ko (1972)
*M. martini*	*R. sanguineus s.l*., *R. turanicus*, *Hyalomma truncatum* (Ixodidae)	Larvae	Larvae, transstadial to nymphs	Nymphs	~11 days	Petit *et al*. (1988); Bain *et al*. (1986)
*Yatesia*						
*Y. hydrochoerus*	*A. cajennense*, *A. americanum* (Ixodidae)	Nymphs	Nymphs, transstadial to adults	Adults	~26 days	Yates & Lowrie (1984)

Filarial nematodes transmitted by ticks can potentially cause mild health effects in vertebrate hosts, primarily manifesting as minor skin alterations (e.g. dermatitis, alopecia) due to the accumulation of microfilariae. While these infections can occasionally lead to subcutaneous nodules, lymphatic changes, or create opportunities for secondary infections, they are most often of limited pathological significance (Otranto *et al*., 2012*b*; Otranto, 2015; Ajileye *et al*., 2025). However, some tick‐borne filarial species can cause significant pathology in their vertebrate hosts. For instance, *Cercopithifilaria johnstoni* infections in their natural hosts (bush rats, *Rattus fuscipes*) and laboratory rats produce cutaneous and ocular immunopathologies resembling those observed in human onchocerciasis (Spratt & Haycock, 1988; Vuong *et al*., 1993). Despite such findings, the full clinical manifestations and impacts of filarial nematode infection on wildlife health and fitness – including potential effects on survival, reproduction, and behaviour in free‐ranging animals – remain poorly understood. In this review, we examine several intrinsic and extrinsic factors that potentially contribute to successful filarial nematode transmission in tick vectors, drawing insights from extensively studied dipteran vector systems. Understanding these processes requires comprehensive empirical data on tick immune responses to filarial infections, how nematodes are transmitted, and how abiotic conditions may influence these interactions. We also examine how host movement across ecological settings, tick life stages, and feeding preferences may contribute to parasite introduction, establishment, and range expansion. By integrating vector biology, parasitology, and ecology, we aim to provide insights into the transmission dynamics of filarial nematodes and the potential impact these parasites may have on tick vectors and vertebrate hosts.

## II. POTENTIAL FACTORS SHAPING TICK‐BORNE FILARIAL NEMATODE TRANSMISSION DYNAMICS

The roles of intrinsic (related to the vector and parasite biology) and extrinsic (related to the environment and ecological context) factors in mosquito‐borne and other fly‐borne filarial nematode transmission have been extensively studied and we expect factors important in dipteran systems also will be important in tick systems. Transmission dynamics in tick‐borne systems may be driven by a complex interplay between intrinsic and extrinsic factors. Critical determinants of transmission dynamics include vector biological factors (tick life cycles, anatomical barriers, and immune responses), parasite biological factors (intraspecific variation, microfilarial periodicity phenomena, and enhancement mechanisms), ecological factors (host movements and ecological interfaces), and environmental conditions (temperature, humidity, and landscape features). These factors potentially interact in complex ways, affecting the success of filarial nematode transmission.

### (1) Intrinsic factors affecting transmission

#### (a) Tick life stages and their role in filarial nematode transmission

**Fig. 1:**
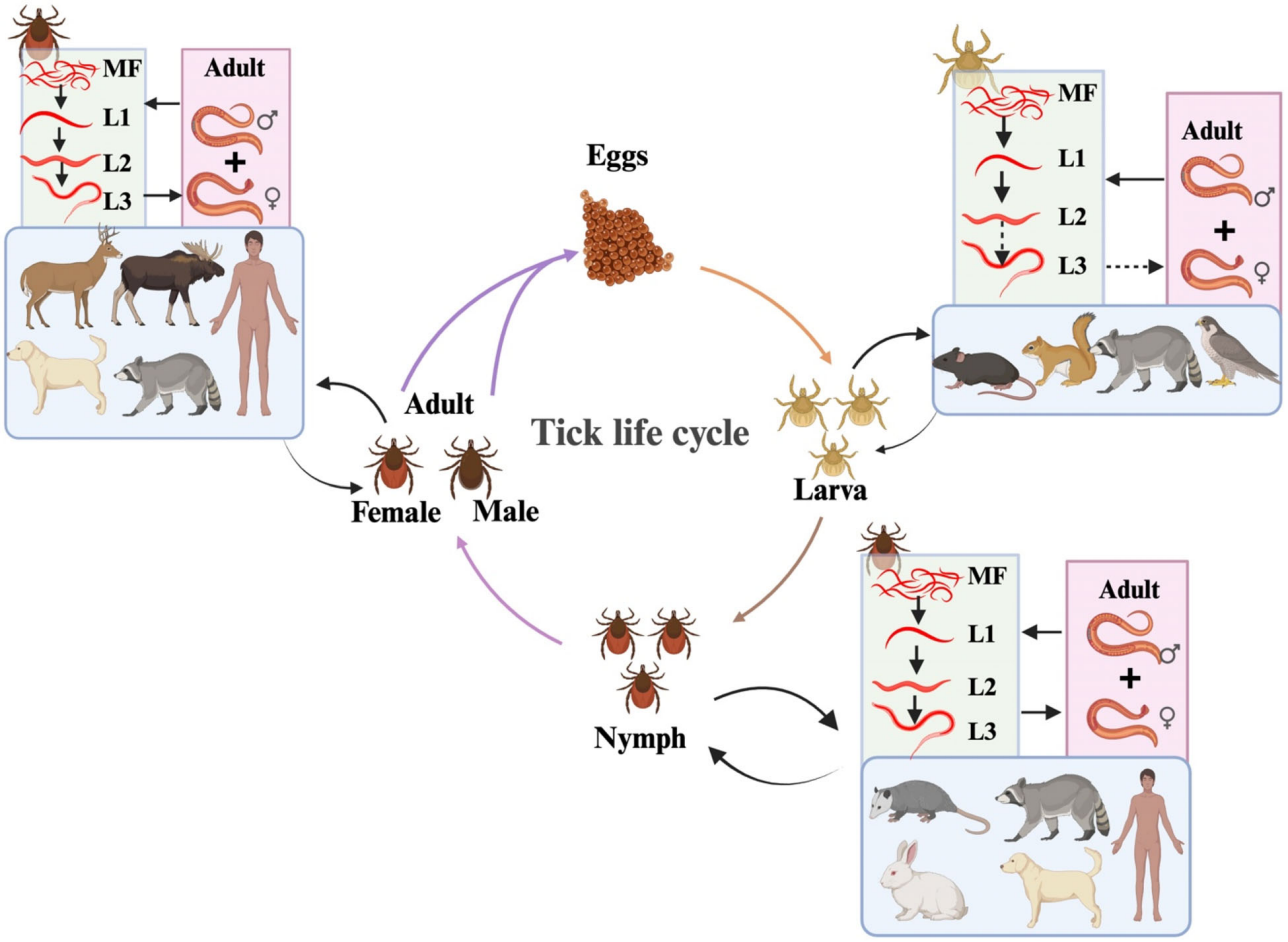
Potential transmission cycle of filarial nematodes with tick vectors. Filarial nematode development in tick vectors (green boxes: microfilariae to L3 stages) and vertebrate hosts (pink boxes: adult nematodes) varies across species. Ticks acquire microfilariae (MF) during blood feeding on infected hosts. The tick stage that acquires, supports development, and transmits the infection differs among filarial species. Some species are acquired by larval ticks and complete development to L3 during the larval–nymph moult, with transmission by nymphs; others are acquired by nymphal ticks, undergo transstadial development, and are transmitted by adults. Larval ticks that acquire microfilariae cannot complete development to the infective L3 stage; development to L3 occurs only after moulting to the nymphal stage. The anatomical site of development also varies: it may take place entirely within the epidermis or within musculature. L3 larvae are transmitted to vertebrate hosts during subsequent feeding, where they mature into adult male (♂) and female (♀) nematodes that produce microfilariae. The three panels illustrate different transmission patterns associated with diverse vertebrate hosts (wildlife, domestic animals, and potentially humans). Solid arrows indicate developmental progression and successful transmission; dotted arrows indicate developmental or transmission steps that cannot occur at that life stage. MF = microfilariae; L1 = first‐stage larvae; L2 = second‐stage larvae; L3 = third‐stage (infective) larvae.

The transmission of filarial nematodes by tick vectors represents a complex biological process that intertwines the life cycles of the tick vector and the vertebrate host (Fig. 1). Tick life stages, which include larvae, one or more nymphal stages (depending on tick family), and adults may play distinct roles in the acquisition, maintenance, and transmission of filarial nematodes, contributing to the overall dynamics of the vector–parasite relationship (Ramos *et al*., 2013, 2013; Uni *et al*., 2013; Bruley & Duron, 2024). Once ingested, microfilariae (first filarial nematode life stage) undergo development in the tick gut, progressing through three larval stages (L1, L2, and L3); the timing of each life stage can vary depending on specific tick–filarial nematode species associations (Otranto *et al*., 2012*a*; Brianti *et al*., 2012; Ramos *et al*., 2013*a*) (Table 1). These timing differences suggest that developmental rates may be influenced by factors such as filarial nematode species, tick species/life stage, host characteristics, feeding duration, and physiological environment.

Tick larvae can acquire filarial nematodes from vertebrate hosts during their prolonged blood feeding, which can be between 3 and 7 days for most Ixodidae ticks (Ko, 1972). Transstadial transmission (transmission of the parasite through the moulting process of an individual tick) is crucial for the survival of these larval‐acquired parasites (Binetruy & Duron, 2023; Ramos *et al*., 2013*b*; Brianti *et al*., 2012). Studies of filarial nematode species *Acanthocheilonema dracunculoides* and *Monanema marmotae* in *Rhipicephalus sanguineus* (*s.l*.; in the broad sense, referring to a species complex) and *Ixodes cookei*, respectively, demonstrate that microfilariae acquired by larval ticks do not complete development until post‐moult to the nymphal stage in these systems (Olmeda‐Garcia & Rodriguez‐Rodriguez, 1994; Ko, 1972). Thus, while microfilariae may survive the tick larval–nymphal moult (Ramos *et al*., 2013*a*; Brianti *et al*., 2012), their development appears to be dependent on the life stage of the tick vector. Microfilariae acquired by larval ticks continue developing during the nymphal stage, but complete maturation to the infective L3 stage is likely stimulated during the nymphal–adult moult (Ko, 1972; Olmeda‐Garcia & Rodriguez‐Rodriguez, 1994; Otranto *et al*., 2012*a*; Brianti *et al*., 2012; Petit *et al*., 1988). Adult ticks transmit L3s during feeding, as documented across multiple tick–filarial systems (Brianti *et al*., 2012; Ramos *et al*., 2013*b*; Ko, 1972), supporting the role of infected blood‐fed nymphs moulting to adults as the required step necessary for filarial transmission in these systems. Adult ticks may be more efficient vectors due to their generally longer attachment periods, which vary by species but typically range from 5 to 11 days, compared to 3–5 days for nymphs (Dantas‐Torres, 2010; Sonenshine & Roe, 2014). This extended feeding time increases opportunities for both acquiring microfilariae and transmitting L3s to vertebrate hosts (Ramos *et al*., 2013*b*; Dantas‐Torres, Chomel & Otranto, 2012; Otranto *et al*., 2013; Winkhardt, 1980). Whether microfilariae acquired by adult ticks during feeding can develop to the infective L3 stage, particularly in species with interrupted or repeated feeding patterns, remains unclear.

#### (b) Tick anatomical barriers to filarial nematode development and transmission

**Fig. 2:**
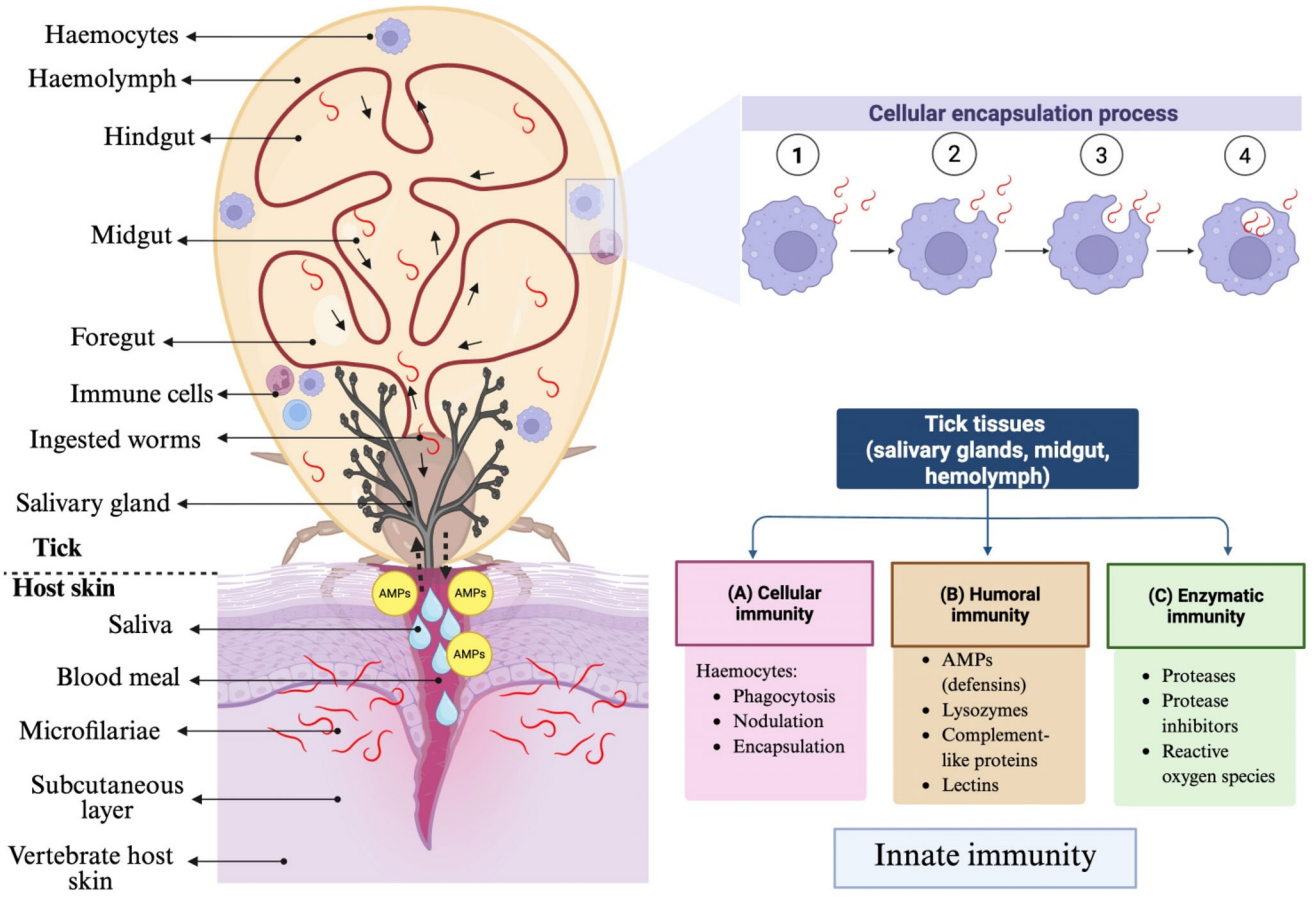
Potential mechanism of tick anatomical defences in filarial nematode interactions. Left panel shows a cross section of an attached tick feeding on vertebrate host skin, illustrating the potential migration paths of microfilariae through various tick tissues. The feeding lesion shows the tick's mouthparts penetrating through the host skin layers, where microfilariae can be ingested with the blood meal. Key anatomical features include the branched salivary glands, the segmented gut system (foregut, midgut, and hindgut), circulating haemocytes, haemolymph, and labelled structures showing where ingested nematodes and immune cells are located. This anatomical organization presents multiple barriers that filarial nematodes must overcome during transmission. Right panel shows the three main components of tick innate immunity: (A) cellular immunity *via* haemocytes performing phagocytosis (for small pathogens), nodulation, and encapsulation, with the cellular encapsulation detailed in steps 1–4 showing how haemocytes may surround larger parasites, like filarial nematodes; (B) humoral immunity involving antimicrobial peptides (AMPs), lysozymes, complement‐like proteins, and lectins; and (C) enzymatic immunity through proteases, protease inhibitors, and reactive oxygen species. All these immune components operate within tick tissues, including salivary glands, midgut, and haemolymph.

Larval stages of filarial nematodes must overcome multiple anatomical barriers within tick vectors from initial ingestion as microfilariae to eventual transmission as L3s. This developmental journey involves significant morphological changes: microfilariae are typically slender (ranging from 3 to 17 μm in width depending on genus and species, with most averaging 3–11 μm) and relatively short (180–670 μm in length), progressing through L1 stages (~190 μm long and 5.5 μm wide) and L2 stages (~798 μm long and 26 μm wide), before finally developing into mature L3 larvae (~1700 μm long and 27 μm wide). These size changes present potential physical challenges when traversing narrow tick anatomical structures like salivary ducts (Otranto *et al*., 2011; Bezerra‐Santos *et al*., 2023; Ko, 1972; Fig. 2). Filarial nematode transmission through tick vectors involves navigating multiple sequential barriers within the body of the tick: first through the gut system, haemocoel, and salivary tissues, and finally the hypostome and mouthparts (Fig. 2; left panel).

The mechanisms used to overcome anatomical barriers within ticks (which affects vector competence) remain largely unknown. Following initial ingestion of the blood meal, microfilariae accumulate within the tick gut system, potentially interfering with digestion before penetrating through the midgut epithelium, and developing to L3s in the haemocoel, from which they migrate to the mouthparts for transmission (Ko, 1972). While microfilariae of filarial species that use mosquitoes as vectors penetrate the midgut within hours using proteolytic enzymes and mechanical force (Bain, 1967; Christensen & Sutherland, 1984), detailed mechanisms in ticks remain unexplored despite observations of midgut traversal (Ko, 1972). The extended tick feeding period (3–11 days depending on tick life stage) makes it challenging to establish precise timeframes for microfilariae development to the infective L3 stage, as individual ticks may contain microfilariae ingested at different timepoints during feeding (Ko, 1972). For example, *M. marmotae* microfilariae have been observed penetrating the gut wall, residing in the haemocoel, and entering epidermal cells within 7 days of tick attachment (Ko, 1972; Bain & Babayan, 2003). After gut penetration, microfilariae must survive in the haemocoel environment while developing through L1 and L2 stages (Ko, 1972; Brianti *et al*., 2012). Their precise migration pathways and survival mechanisms remain unknown. Notably, development to the L3 stage in *Cercopithifilaria bainae* takes 25–30 days in ticks (Brianti *et al*., 2012) compared to 8–10 days for mosquito‐borne filarial species like *Brugia malayi* (Erickson *et al*., 2009), which could reflect species‐level differences, vector‐specific survival strategies, or simply inherent differences in development rates between tick‐borne and mosquito‐borne filarial species.

Infective larvae (L3) have been documented in specific anatomical locations of tick salivary glands: *C. bainae* within salivary gland acini of *R. sanguineus s.l*. and *M. marmotae* in salivary gland ducts of *Ixodes cookei* (Brianti *et al*., 2012; Ko, 1972). Bain (1967) found *Acanthocheilonema viteae* L3 larvae in both the pharynx and salivarium of *Ornithodoros tartakovskyi*. These studies suggest larval penetration of these structures during tick feeding. The salivary glands serve as critical sites for pathogen transmission to vertebrate hosts (Šimo *et al*., 2017). Salivary glands can produce compounds essential for modulating host immune responses, inflammation, and haemostasis during blood feeding (Francischetti *et al*., 2009), all of which can affect pathogen transmission. Tick salivary glands also secrete immunomodulatory factors and anticoagulant molecules that could influence pathogen survival and transmission (Kazimírová & Štibrániová, 2013; Francischetti *et al*., 2009). While salivary gland localization is common for many tick‐borne pathogens, filarial L3 larvae may utilize alternative transmission routes. For example, in mosquito systems, *Dirofilaria immitis* L3 reside in mouthparts rather than salivary glands (Šimo *et al*., 2017; Hajdušek *et al*., 2013), while in mite vectors, *Litomosoides* species have been documented in interstitial tissue and coxal glands in addition to salivary glands (Diagne *et al*., 1989). Additionally, the costs of filarial infection on vector fitness have been documented in mite vectors, where *L. sigmodontis* infections negatively affect mite survival and reproduction (Nieguitsila *et al*., 2013). This suggests that similar fitness trade‐offs may occur in tick–filarial systems as ticks mount immune responses against developing nematodes.

Other tick‐borne pathogens like *Borrelia burgdorferi* have shown preferential movement towards and colonization of salivary glands (tissue tropism) through pathogen surface protein–tick receptor interactions (Pal *et al*., 2004); the specific mechanisms of entry, survival, and transmission in tick salivary glands remain largely unknown for filarial nematodes. Studies of mosquito‐borne filarial nematodes suggest that larvae use chemotactic signals to navigate within vector tissues (Wheeler *et al*., 2020). In ticks, microfilariae have been observed accumulating at tick attachment sites on the vertebrate host (Ko, 1972; Moorhouse, 1969), potentially responding to chemical cues from tick saliva or the feeding lesion. L3s are typically found in the haemocoel before actively migrating to tick mouthparts for transmission to vertebrate hosts, suggesting a directed migration pattern that may involve similar chemotactic mechanisms (Bain & Babayan, 2003). The absence of transovarial transmission is likely due to anatomical incompatibility, as the relatively large size of developing filarial larvae would prevent passage through the narrow oviducts and other reproductive structures of female ticks. Similar coordination between parasite development and vector feeding occurs in mosquito‐borne systems, such as *Brugia malayi* where microfilariae penetrate the midgut of the vector within 1–2 h of ingestion and time their development to coincide with subsequent blood meals (Erickson *et al*., 2009). This may also be the case for tick‐borne filarial nematodes.

#### (c) Tick immune responses

The successful transmission of tick‐borne pathogens, including filarial nematodes, depends on their ability to evade tick immune defences (Fig. 2, right panel). Ticks rely on innate immunity, comprising cellular, humoral, and enzymatic components, to combat invading pathogens (Hajdušek *et al*., 2013). Immunity at the cellular level is mediated by tick haemocytes, circulating immune cells that function similarly to vertebrate white blood cells (Hajdušek *et al*., 2013). These haemocytes perform immune functions including phagocytosis (the cellular process of engulfing and destroying foreign particles), immune factor production, and encapsulation (the surrounding and isolation of large foreign bodies) (Hajdušek *et al*., 2013; Rolandelli *et al*., 2024; Fogaça *et al*., 2021). Studies have shown encapsulation and phagocytosis to combat microbial infections effectively in dipteran insects and ticks through the formation of multilayered cellular capsules that isolate and kill pathogens (Rolandelli *et al*., 2024). However, their role against macroparasites, like filarial nematodes in tick vectors, remains unknown. The humoral immune response in ticks involves soluble factors in haemolymph, primarily antimicrobial peptides (AMPs) that directly kill or inhibit pathogen growth. Ticks produce several AMP families, including defensins (Kopáček *et al*., 2010; Hajdušek *et al*., 2013; Wu *et al*., 2022), and employ other factors such as complement‐like proteins, proteases, and lectins that recognize specific pathogen surface structures (Pal *et al*., 2004; Fogaça *et al*., 2021).

While our understanding of evasion strategies in tick–filarial nematode relationships is lacking, insights can be drawn from other nematode–arthropod systems. Research has shown that pathogens employ various molecular and physiological mechanisms, including surface protein variations that affect immune evasion and tissue adherence (as shown in *B. burgdorferi*; Kenedy, Lenhart & Akins, 2012; Doskaliuk & Zimba, 2024), differences in metabolic adaptation to tick environments (Hajdušek *et al*., 2013), and variations in developmental timing (Otranto *et al*., 2013). Mosquito‐borne filarial nematodes have several evasion strategies to suppress the immune defence of mosquito vectors through anatomical seclusion, camouflage, and interference strategies (Castillo, Reynolds & Eleftherianos, 2011; Erickson *et al*., 2009). Another potential evasion strategy includes molecular mimicry, where parasites present surface molecules resembling host proteins to avoid immune recognition (Hurford & Day, 2013), and active suppression of vector immunity through secreted immunomodulatory molecules (Loghry *et al*., 2023; Erickson *et al*., 2009). Research has also highlighted the role of extracellular vesicles (EVs) and microRNAs in parasite–host interactions. Filarial nematodes secrete EVs containing various bioactive molecules, including microRNAs (small non‐coding RNA molecules that regulate gene expression), that can manipulate host immune responses and alter gene expression in host cells (Loghry *et al*., 2023; Zamanian *et al*., 2015; Tritten *et al*., 2014). These small RNA regulators represent a sophisticated cross‐species communication and immune‐modulation mechanism. Additionally, filarial nematodes may employ antioxidant enzymes like superoxide dismutase and catalase to neutralize reactive oxygen molecules produced during immune responses (Selkirk *et al*., 1998; Henkle‐Dührsen & Kampkötter, 2001; Hotterbeekx *et al*., 2021). The developmental stage of filarial nematodes may also influence their immune interactions, with microfilariae showing greater resistance to immune responses compared with later stages. This enhanced resistance stems from unique surface properties and molecular mechanisms that enable microfilariae to evade host immune recognition during their development within the vector (Le Goff *et al*., 2000; Moreno & Geary, 2008). Later within‐vector developmental stages may face more complex immunological interactions, potentially increasing their vulnerability to immune responses.

#### (d) Intraspecific variation and host specificity adaptations

Genetic and phenotypic variation within nematode species (intraspecific variation) can complicate host specificity patterns in filarial nematode populations. While this phenomenon has been well documented in mosquito‐borne systems, comparable data for tick‐borne filarial nematodes remain largely absent. For example, in mosquito‐borne filarial nematodes, different genetic strains of *Wuchereria bancrofti* show varying abilities to develop in different vectors – some strains develop successfully in *Culex* mosquitoes but poorly in *Anopheles* species, while other strains show the opposite pattern (Pichon, 2002; Paily, Hoti & Das, 2009). Similar strain‐specific patterns occur in tick‐borne pathogens. For instance, distinct genetic lineages of *B. burgdorferi* and *Anaplasma phagocytophilum* vary in their ability to survive and multiply within different *Ixodes* species, resulting in different infection outcomes (Crowder *et al*., 2010; Hanincová *et al*., 2008; Lesiczka *et al*., 2023; Rejmanek, Bradburd & Foley, 2012). These genetic differences also affect pathogenicity; for example, deer‐associated strains of *A*. *phagocytophilum* are not pathogenic to humans, while strains associated with other reservoir hosts can cause human granulocytic anaplasmosis (Foley *et al*., 2008). While intraspecific variation in tick‐borne filarial nematodes remains undocumented, distinct species within the same genus are associated with specific host–vector combinations. The genus *Cercopithifilaria* illustrates this pattern: *C*. *bainae* in dogs (*Canis familiaris*) is transmitted by *R. sanguineus s.l*., *C*. *rugosicauda* in roe deer (*Capreolus capreolus*) is transmitted by *Ixodes ricinus*, and *C. grassi* in dogs is also transmitted by *R. sanguineus s.l*. (Otranto *et al*., 2013; Bezerra‐Santos *et al*., 2022). The limited documentation of genetic and morphological variation in tick‐borne filarial nematodes may reflect their infrequent detection rather than actual biological patterns (Bezerra‐Santos *et al*., 2022).

#### (e) Potential microfilarial periodicity and enhancement in tick‐borne pathogens

Two significant phenomena observed in non‐tick vector‐borne filarial transmission – microfilarial periodicity and enhancement – remain unexplored in tick‐borne filarial systems. Microfilarial periodicity is the cyclical fluctuation in microfilariae concentration within vertebrate host blood and tissues, regulated by circadian rhythms and physiological cues. Variation in microfilaria periodicity follows predictable 24‐h cycles synchronized with vector feeding patterns and host activities (Hawking, 1967; Aoki, Fujimaki & Tada, 2011). For instance, studies on mosquito‐borne filariasis show that *W. bancrofti* exhibits nocturnal periodicity in regions with night‐biting mosquitoes, while *B. malayi* shows nocturnal sub‐periodicity, maintaining some presence during daylight hours while peaking at night (Pichon, 2002). This synchronized timing maximizes the likelihood of microfilaria being ingested by feeding vectors (Schneider *et al*., 2018). The dynamics of periodicity in tick‐borne systems, if present, are likely to differ due to fundamental differences in vector feeding behaviour. Unlike mosquitoes, which feed for minutes, hard ticks remain attached to hosts for days to weeks (Sonenshine & Roe, 2014; Ajileye *et al*., 2025). The extended attachment period of ticks suggests that classical periodicity patterns observed in mosquito‐borne filarial systems may be less relevant for tick‐borne transmission. Instead, microfilarial availability might be influenced by different phases of tick engorgement rather than host circadian activity patterns.

Studies on dermal filarial nematodes of rodents, including *Litomosoides sigmodontis* transmitted by mites and *A. viteae* transmitted by ticks, do not show regular circadian rhythms (i.e. are non‐periodic) (Hawking, 1967). Instead, when rodent hosts retreat to burrows, the resulting drop in environmental temperature triggers microfilariae to swarm to sites, usually dermal tissues (many tick‐borne filarial nematodes, including but not limited to the *Dipetalonema* group, primarily reside in the dermal tissues rather than circulating in peripheral blood; Otranto *et al*., 2012*b*, 2013), where they become available to arthropod vectors (Hawking, 1967; Hawking *et al*., 1967; Hawking & Thurston, 1951). This temperature‐dependent rather than time‐dependent periodicity suggests adaptation to host behaviour and microhabitat, which may also apply to tick‐borne filarial systems. Additional evidence supporting specialized periodicity patterns partially exists in several studies. Moorhouse (1969) observed microfilariae accumulating beneath *Ixodes tasmani* attachment sites, suggesting that tick feeding triggers localized microfilarial recruitment. Similarly, Ko (1972) documented that microfilarial concentration increases near tick feeding sites, partly due to chemical attraction (dermotropic signals) to salivary deposits during feeding. These observations indicate a potential site‐specific periodicity that differs from the systemic, time‐dependent periodicity observed in mosquito‐borne systems.

**Fig. 3:**
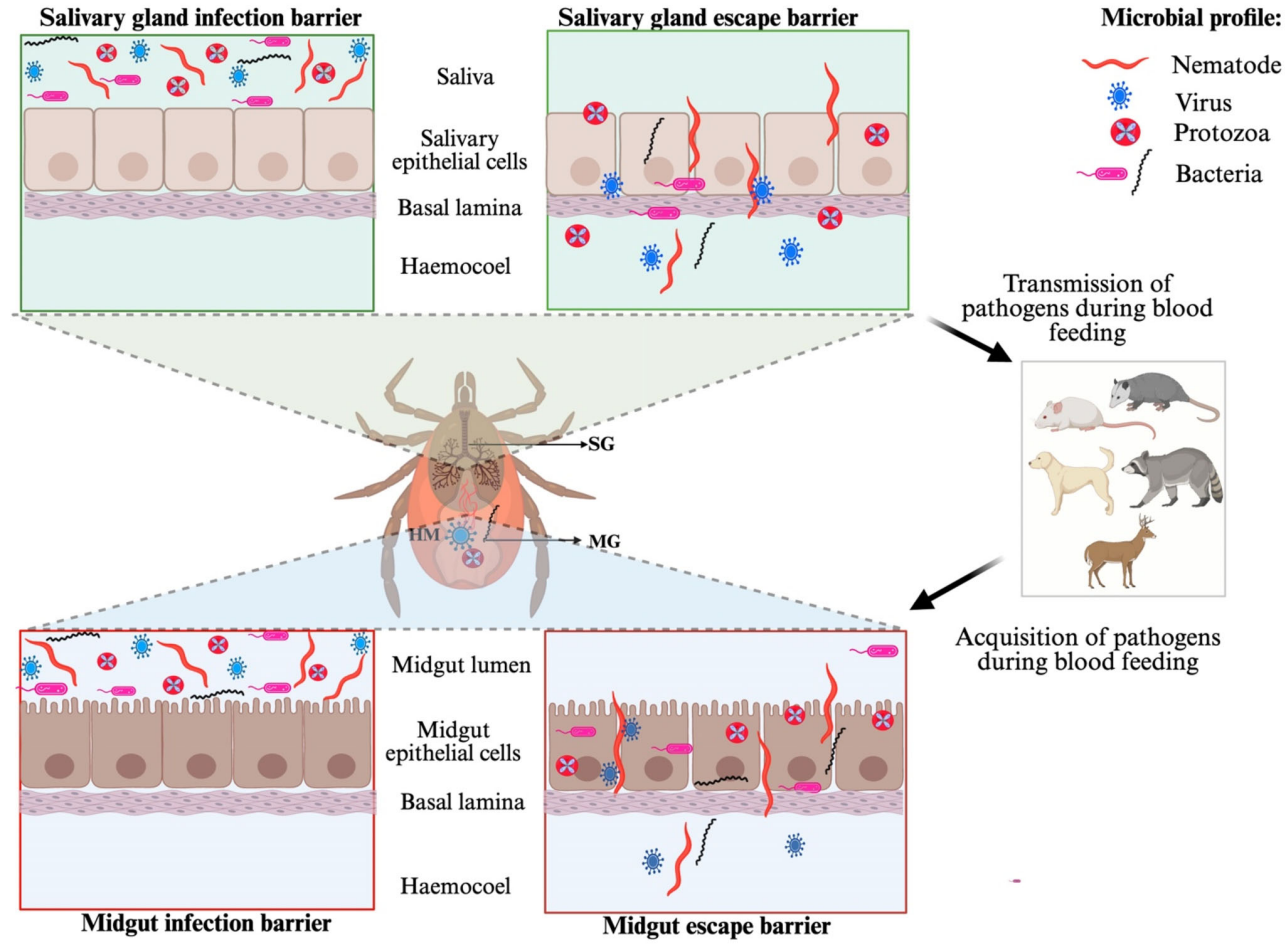
Anatomical barriers and pathogen migration in tick vector transmission. Four critical barriers that pathogens must navigate within tick vectors: midgut (MG) infection barrier, midgut escape barrier, salivary gland (SG) infection barrier, and salivary gland escape barrier. Filarial nematodes penetrate the midgut epithelium creating micro‐lesions that may facilitate passage of co‐infecting pathogens (viruses, protozoa, bacteria) in a process known as enhancement. This phenomenon potentially accelerates the transmission of tick‐borne pathogens that would otherwise be limited by anatomical barriers. The tissue cross‐sections show epithelial cells overlying the basal lamina, with haemocoel (HM) beneath. Diverse vertebrate hosts shown represent animals involved in pathogen acquisition and transmission.

Microfilarial enhancement refers to how microfilariae facilitate the dissemination of other pathogens across barriers within arthropod vectors (Vaughan & Turell, 2017) (Fig. 3). In mosquito systems, microfilariae penetrate the midgut epithelium using cephalic hooks and proteolytic enzymes, creating micro‐lesions that breach the midgut barrier. These perforations serve as pathways through which viruses, bacteria, or protozoa can pass from the blood meal into the haemocoel, bypassing barriers that typically prevent their dissemination and conferring vector competence on otherwise incompetent vectors (Fig. 3). Infectious pathogens benefiting from microfilarial enhancement demonstrate shortened extrinsic incubation periods, allowing them to infect and escape salivary glands more rapidly, resulting in earlier and more efficient transmission to vertebrate hosts (Vaughan & Turell, 2017) (Fig. 3). This ‘leaky gut’ phenomenon has been demonstrated for several virus–filarial nematode–mosquito systems, including West Nile virus and Rift Valley fever virus (Turell, Gargan & Bailey, 1984; Vaughan *et al*., 2012). If similar mechanisms exist in ticks, microfilarial‐induced perforations could potentially accelerate transmission timelines or turn non‐vectors into vectors for various tick‐borne pathogens, which would have significant epidemiological implications for disease spread.

### (2) Extrinsic factors affecting transmission

#### (a) Ecological factors

##### (i) Tick–host relationships and movement‐mediated transmission

Ticks exhibit either one‐host, two‐host, or three‐host life cycles. One‐host ticks, such as *Rhipicephalus* (*Boophilus*) *microplus*, remain on the same host (e.g. cattle, *Bos taurus*) for all three life stages: larva, nymph, and adult (Leal, Thomas & Dearth, 2018; Labruna *et al*., 2009). Two‐host ticks, like *Hyalomma marginatum*, use two hosts, typically moulting from larva to nymph on the first host, then seeking a second host as adults. Three‐host ticks, including medically important species like *Ixodes scapularis* and *Dermacentor variabilis*, seek a new host for each life stage (Leal *et al*., 2020; Hamer *et al*., 2012). Compared to one‐ and two‐host tick species, three‐host ticks may have more opportunities to acquire microfilariae from infected vertebrate hosts.

Tick–filarial nematode systems are believed to exhibit high host specificity, creating specialized transmission pathways despite the broader feeding patterns of some tick vectors. Even ticks that feed on multiple host species seem to transmit filarial nematodes successfully only to specific vertebrate hosts. For example, although *I. ricinus* feeds on various mammals, birds, and reptiles, *C. rugosicauda* transmission occurs almost exclusively in roe deer (Ramos *et al*., 2013*b*; Winkhardt, 1980). Similarly, while *R. sanguineus s.l*. feeds predominantly on domestic dogs and only occasionally parasitizes other mammalian hosts, transmission of *C. bainae* has been documented exclusively in dogs, suggesting high host specificity of this filarial nematode (Bezerra‐Santos *et al*., 2022; Otranto *et al*., 2013). Such host–parasite specificity demonstrates that successful establishment and biological transmission require compatibility factors beyond mere exposure during tick feeding. Alternatively, it may be that potential vertebrate hosts of filarial nematodes are grossly understudied, and it is unknown if filarial species can be successfully transmitted to a broader host community. Variable tick–host associations result in different tick species coming into contact with different vertebrate hosts, which can create novel transmission networks for tick‐borne filarial nematodes.

Host movement facilitates the geographic dispersal and genetic exchange of ticks, and therefore the filarial nematodes they carry, across multiple environments. Ticks, being relatively immobile, rely on host movements to colonize new habitats and expand their geographic range (Léger *et al*., 2013; Diuk‐Wasser, VanAcker & Fernandez, 2021; Ogden, Mechai & Margos, 2013). These movements can influence tick–pathogen associations across multiple scales. Seasonal bird migrations can transport ticks and their associated pathogens over vast, intercontinental distances (Ogden *et al*., 2008; Hasle, 2013), while mammalian movements affect tick acquisition and distribution of filarial nematodes through daily foraging and home range activities (Ramos *et al*., 2013*b*; Henning *et al*., 2016). However, dispersal must be followed by establishment in local tick populations to maintain transmission cycles. Human activities could influence transmission through international pet trade, tourism with pets, and livestock transportation. For example, the worldwide distribution of *R. sanguineus s.l*., a competent vector for *C. bainae*, has been facilitated by the global movement of dogs through adoption, trade, and travel (Gruntmeir *et al*., 2023; Dantas‐Torres & Otranto, 2014).

##### (ii) Potential transmission dynamics across ecological interfaces

The boundary between natural and human‐modified environments represents a critical zone for parasite exchange, where parasites can move between different ecological settings through tick vectors and host movements. These interfaces create opportunities for transmission between sylvatic (natural, undisturbed environments), domestic (areas with livestock and pets), and urban (city) environments. For example, human encroachment into sylvatic habitat enables parasite exchange from wildlife reservoirs to domestic hosts through shared tick vectors like *Rhipicephalus* and *Ixodes* species (Maia *et al*., 2017; Bezerra‐Santos *et al*., 2022). *I. ricinus* ticks, which feed on various hosts including rodents, birds, and large mammals, can acquire *C. rugosicauda* from roe deer and potentially transmit it to domestic animals at these interfaces (Winkhardt, 1980; Ramos *et al*., 2013b). Similarly, in South America, capybaras (*Hydrochaerus hydrochaeris*) infected with *Cruorifilaria tuberocauda* and *Yatesia hydrochoerus* can facilitate transmission between sylvatic and domestic environments when they inhabit areas bordering human settlements, through tick vectors such as *Amblyomma romitii* and the *Amblyomma cajennense* species complex (Yates & Lowrie, 1984; Vicente *et al*., 1997; Eberhard, Morales & Orihel, 1976; Binetruy & Duron, 2023). These same capybara populations serve as important reservoirs for other tick‐borne pathogens, including *Rickettsia* species within the spotted fever group (Brazilian spotted fever), transmitted by *Amblyomma dubitatum* and *Amblyomma sculptum* throughout South America, highlighting the complex role of capybaras in maintaining multiple pathogen cycles at the wildlife–human interface (Rosa‐Xavier *et al*., 2025; Quadros *et al*., 2021).

At the urban–sylvatic interface, *R. sanguineus s.l*. thrives in urban settings, maintaining *Cercopithifilaria* species transmission among dogs while potentially extending to urban‐adapted wildlife frequenting city parks. In North America, this includes coyotes (*Canis latrans*) and raccoons (*Procyon lotor*), while in Europe, urban foxes (*Vulpes vulpes*) may serve similar roles. In South America, urban‐adapted capybaras in city parks (Serra‐Medeiros *et al*., 2021) create transmission opportunities between urban and sylvatic environments through their associated tick vectors (epidemiologically important reservoirs for spotted fever *Rickettsia*). These complex ecological relationships are facilitated by human activities, including pet ownership, recreational activities in natural areas, and land use changes that create new transmission interfaces. Additionally, global travel and trade enable long‐distance transport of infected pets (and wildlife) and their ticks, potentially expanding filarial nematode distribution across ecological gradients. However, these ecological transmission dynamics remain largely theoretical, as tick‐borne filarial nematode infections occurring simultaneously across connected ecological settings have not been systematically studied (Maia *et al*., 2017). Field investigations documenting simultaneous infections would be necessary to validate these proposed transmission patterns.

#### (b) Environmental factors: temperature and humidity effects

The transmission dynamics of tick‐borne pathogens can be influenced by extrinsic factors such as temperature and humidity. These environmental conditions exert varying effects on tick vectors (and tick‐borne pathogens such as filarial nematodes), shaping geographic distributions, seasonal patterns of tick activity, host‐seeking behaviour, development rates, and overall transmission efficiency. Temperature is likely a primary driver, impacting tick biology and potentially nematode development within tick vectors. For ticks, warmer temperatures generally accelerate development and increase activity, leading to more frequent host‐seeking behaviour and transmission opportunities (Ogden *et al*., 2004; Rodgers, Zolnik & Mather, 2007; Alasmari & Wall, 2021; MacDonald, 2018; Léger *et al*., 2013). However, this relationship is not strictly linear, as extremely high temperatures can also be detrimental to tick survival, particularly in arid conditions (Estrada‐Peña, Ayllón & de la Fuente, 2012). For filarial nematodes, higher temperatures tend to accelerate larval development, potentially shortening the extrinsic incubation period to the L3 stage in arthropod vectors (Fortin & Slocombe, 1981). Climate change‐related studies on mosquito‐borne filarial diseases have projected expanded transmission seasons and potential range shifts for diseases like lymphatic filariasis and onchocerciasis (Kulkarni *et al*., 2016; Lau *et al*., 2016), suggesting similar impacts could occur for tick‐borne filarial systems.

Humidity represents another factor that can affect tick and filarial nematode survival. Ticks are particularly susceptible to desiccation during off‐host periods, with low humidity reducing survival rates and activity levels (Randolph & Storey, 1999; Brunner *et al*., 2023). Under low‐humidity conditions, ticks descend to moist microhabitats near the soil surface to rehydrate, which reduces host‐seeking time and limits their spatial distribution (Medlock *et al*., 2013). When ticks are attached to hosts that move through different microclimatic zones, the attached ticks experience varying humidity conditions, potentially influencing their internal physiology and the survival of ingested microfilariae. The interplay between temperature and humidity likely creates complex ecological niches that may determine the geographic distribution and seasonal activity of tick vectors and potentially filarial nematode transmission. Climate change could alter these patterns by shifting the geographical range of ticks and their filarial nematodes, leading to new transmission dynamics (Medlock *et al*., 2013; Ogden *et al*., 2014). This has already been observed with the northward expansion of *I. scapularis* into Canada, which has been accompanied by the emergence of Lyme disease and anaplasmosis in previously unaffected regions (Ogden *et al*., 2014; Bouchard *et al*., 2019). A similar pattern could potentially occur for tick‐borne filarial nematodes following the range expansion of their tick vectors. Other environmental variables, such as photoperiod, altitude, and landscape features, may also play significant roles in filarial nematode transmission. For example, photoperiod influences tick diapause patterns and seasonal activity (Belozerov, 1982), altitude affects temperature and humidity profiles creating vertical gradients in transmission risk (Daniel *et al*., 2003), and landscape features such as mountains or valleys modulate local microclimates, influencing tick (and filarial nematode) survival and activity patterns (Estrada‐Peña, 2001).

## III. CONCLUSIONS

(1) This review highlights critical knowledge gaps in tick‐borne filarial nematode transmission that warrant systematic investigation. Despite global documentation of these parasites, fundamental aspects of their biology remain unexplored. Understanding microfilarial periodicity and density patterns in dermal tissues, particularly whether site‐specific accumulation occurs during tick feeding, represents a crucial research priority. Molecular characterization of mechanisms enabling filarial nematodes to penetrate tick anatomical barriers and evade immune responses requires urgent attention, as does investigation of potential enhancement phenomena where filarial nematodes may facilitate transmission of co‐infecting pathogens. Such studies could reshape our understanding of tick‐borne disease ecology.
(2) Methodological advances are essential for progress in this field. Development of standardized protocols for detecting and quantifying filarial infections in ticks and vertebrate hosts would enable comparative studies across regions. Advanced imaging techniques could visualize nematode migration pathways through tick tissues, while comparative genomics between successful and unsuccessful tick–parasite associations may identify key compatibility factors. *In vitro* tick tissue models would enable controlled studies of host–parasite interactions that are currently impossible with existing methods.
(3) As climate change alters the geographic distributions and phenology of ticks and their vertebrate hosts, understanding environmental thresholds for nematode development will become increasingly critical. Systematic field surveys across ecological gradients – from sylvatic to urban environments – are needed to document infection prevalence and transmission patterns. The role of host specificity of filarial nematodes to both vertebrate hosts and tick vectors, requires clarification through experimental transmission studies.
(4) The complex interplay between intrinsic factors (anatomical barriers, immune responses, parasite adaptations) and extrinsic factors (host movements, ecological interfaces, environmental conditions) creates a multifaceted transmission system that challenges traditional approaches to vector‐borne disease research. Advancing our understanding requires integrating perspectives from molecular biology, ecology, and environmental science. Only through such interdisciplinary approaches can we fully elucidate the transmission dynamics of these neglected yet potentially important parasites and develop evidence‐based strategies for their management in changing global environments.

## CONFLICTS OF INTEREST

None of the authors have a conflict of interest to disclose.

## Data Availability

Data sharing not applicable to this article as no datasets were generated or analysed during the current study.
